# Supplementary material for: Heat adaptation of phage T7 under an extended genetic code
Source: Virus Evol. 2021 Dec 1;7(2):veab100. doi: 10.1093/ve/veab100 (PMC8923235; doi:10.1093/ve/veab100)
Supplement: veab100_Supp [file veab100_supp.zip › Sequencing_Analysis.zip!/Sequencing_Analysis/A3/evidence/MC_SIDE_2_49.html]

BRESEQ :: Evidence


| Missing coverage evidence... | | | | | | | | | | |
| --- | --- | --- | --- | --- | --- | --- | --- | --- | --- | --- |
|  |  |  | seq id | start | end | size | ←reads | reads→ | gene | description |
| \* | \* | ÷ | NC\_001604.1 | 1 | 110 | 110 | NA [0] | [1055] 1062 | *–/–* | –/– |

Only 100 of 1062 total aligned reads displayed.  

`‑‑‑‑‑‑‑‑‑‑‑‑‑TCTCACAGTGTACGGACCTAAAGTT‑CCCCCATA‑GGGGGTACCTAAAGCCCAGCCAATCACCTAAAGTCAACCTTCGGTTGACCTTGAGGGTTCCCTAAGGGTTGGGGATGACCCTTGGGTTTGTCTTTGGGTGTTACCTTGAGTGTCTCTCTGTG‑T‑CC‑CTATCTGTTACAGTCTCCTAAAGTATCCTCCTAAAGTCACCTCCTAACGTCCATCCTAAAGCCAACACCTAAAGCCTACACCTAAAGACCCATCAAGTCAACGCCTATCTTAAAGTTTAAACA‑‑‑‑‑‑‑‑‑‑‑‑‑TAAAGACCAGACCTAAAGACCAGACCTAAAGACACTACATAAAGACCAGACCTAAAGACGCCTTGTTGTTAGCCATAAAGTGATAACCTTTAATCATTGTCTTTATTAATACAACTCACTATAAGGAGAG  >  NC_001604.1/1‑411`  

                                                                                                                             `|`                                                                                                                                                                                                                                                                                                                              
`gggctcggagatgtgtataagagacaggACCTAAAGTT‑CCCCCATA‑GGGGGTACCTAAAGCCCAGCCAATCACCTAAAGTCAACCTTCGGTTGACCTTGAGGGTTCCCTAAGGGTTGGGGATGACCCTTGGGTTTGTCTTTTGGTGTTACCTTGAGTGTCTCTCTGTG‑T‑CC‑C‑ATCTGTTACAGTCTCCTAAAGTATCCTCCTAAAGTCACCTCCTAACGTCCATCCTAAAGCCAACACCTAAAGCCTACACCTAAAGACCCATCAAGTCAACGCCTATCTTAAAGTTTAAACA‑‑‑‑‑‑‑‑‑‑‑‑‑taaagacc                                                                                                                            <  1:261288/275‑1 (MQ=25)`  
`cggagatgtgtataagagacaggtACGGACCTAAAGTT‑CCCCCATA‑TGGGGTACCTAAAGCCCAGCCAATCACCTAAAGTCAACCTTCGGTTGACCTTGAGGGTTCCCTAAGGTTTGGGGATGACCCTTGGGTTTGTCTTTTGGTGTTACCTTGAGTGTCTCTCTGTG‑T‑CC‑CTATCTGTAACAGTCTCCTAAAGTATCCTCCTAAAGTCACCTCCTAACGTCCATCCTAAAGCCAACACCTAAAGCCTACACCTAAAGACCCATCAAGTCAACGCCTATCTTAAAGTTTAAACA‑‑‑‑‑‑‑‑‑‑‑‑‑taaagac                                                                                                                             <  1:7549/279‑1 (MQ=25)`  
 `ggctgggaggtggcaatacgagacagtaCCTAGCGTC‑CCCCCATA‑GGAGGTACCTAATGCCCAGCTAATCACCTAGAGTCAACCTTCGGTTGACCTTGAGGGTTCACTAAGGGTTGGGGATGACCCTTGGGTTTGTCTTTGGGTGTTACCTTGAGTGTCTCTCTGTG‑T‑CC‑CTATCTGTAACAGTCTCCTAAAGTAGCCTCATAAGGTCACCTCCTAACGTCCATCCTAATGCCAACACCTAAAGCCTACACCTAAAGACTCATCAAGTCAACGCCTATCTTAAAGTTTACACA‑‑‑‑‑‑‑‑‑‑‑‑‑taaagacc                                                                                                                            <  1:245827/274‑1 (MQ=21)`  
  `gatgtgtataagagacaggtgtACGGACCTAAAGTT‑CCCCCATA‑GGGGGTACCTAAAGCCCAGCAAATCACCTAAAGTCAACCTTCGGTTGACCTTGAGGGTTCCCTAAGGGTTGGGGATGACCCTTGGGTTTGTCTTTGGGTGTTACCTTGAGTGTCTCTCTGTG‑TCCC‑CTATCTGTTACAGTCTCCTAAAGTATCCTCCTAAAGTCACCTCCTAACGTCCATCCTAAAGCCAACACCTAAAGCCTACACCTAAAGACCCATCAAGTCAACGCCTATCTTAAAGTTTAAACA‑‑‑‑‑‑‑‑‑‑‑‑‑taaagacc                                                                                                                            <  1:61029/283‑1 (MQ=255)`  
       `gtgtataagagacaggtACGGACCTAAAGTT‑CCCCCATA‑GGGGGTACCTAAAGCCCAGCCAATCACCTAAAGTCAACCTTCGGTTGACCTTGAGGGTTCCCTAAGGGTTGGGGATGACCATTGGGTTTGTCTTTGGGTGTTACCTTGAGTGTCTCTCTGTG‑TCCC‑CTATCTGTTACAGTCTCCTAAAGTATCCTCCTAAAGTCACCTCCTAACGTCCATCCTAAAGCCAACACCTAAAGCCTACACCTAAAGACCCATCAAGTCAACGCCTATCTTAAAGTTTAAACA‑‑‑‑‑‑‑‑‑‑‑‑‑taaagaccagacc                                                                                                                       <  1:169832/286‑1 (MQ=255)`  
              `agacaggtgtACGGACCTAAAGTT‑CCCCCATA‑GGGGGTACCTAAAGCCCAGCCAATCACCTAAAGTCAACCTTCGGTTGACCTTGAGGGTTCCCTAAGGGTTGGGGATGACCCTTGGGTTTGTCTTTGGGTGTTACCTTGAGTGTCTCTCTGTG‑T‑CC‑CTATCTGTAACAGTCTCCTAAAGTATCCTCCTAAAGTCACCTCCTAACGTCCATCCTAAAGCCAACACCTAAAGCCTACACCTAAAGACCCATCAAGTCAACGCCTATCTTAAAGTTTAAACA‑‑‑‑‑‑‑‑‑‑‑‑‑taaagaccagacctaaagacc                                                                                                               <  1:166413/295‑1 (MQ=255)`  
                   `ggtgtACTGACCTAAAGTT‑CACCCATA‑GGGGGTACCTAAAGCCCAGCCAATCACCTAAAGTCAACCTTCGGTTGACCTTGAGTGTTCCCTAAGGGTTGGGGATGACCCTTGGGTTTGTCTTTGGGTGTTACCTTGAGTGTCTCTCTGTG‑T‑CC‑CTATCTGTAACAGTCTCCTAAAGTATCCTCCTAAAGTCACCTCCTAACGTCCATCCTAAAGCCAACACCTAAAGCCTACACCTAAAGACCCATCAAGTCAACGCCTATCTTAAAGTTTAAACA‑‑‑‑‑‑‑‑‑‑‑‑‑taaagaccagacctaaagaccagacc                                                                                                          <  1:110227/300‑1 (MQ=255)`  
                    `gtgtATGGACCTAAAGTT‑CCCCCATA‑GGGGGTACCTAAAGCCCAGCCAATAACCTAAAGTCAAACTTCGGTTGACCTTGAGGGTTCCCTAAGGGTGGGGGATGACCCTTGGGTTTGTCTTTGGGTGTTACCTTGAGTGTCTCTCTGTG‑T‑CC‑CTATCTGTAACAGTCTCCTAAAGTATCCTCCTAAAGTCACCTCCTAACGTCCATCCTAAAGCCAACACCTAAAGCCTACACCTAAAGACCCCTCAAGTCAACGCCTATCTTAAAGTTTAACCA‑‑‑‑‑‑‑‑‑‑‑‑‑Taaaaaccagacctaacgcccagacct                                                                                                         >  1:43706/1‑301 (MQ=255)`  
                    `gtgtACGGACCTAAAGTT‑CCCCCCTA‑GGGGGGACCTAAAGCCCAGCCAATCACCTAAAGTCAACCTTCGGTTGACCTTGAGGGTTCCCTAAGGGTTGGGGATGACCCTTGGGTTTGTATTTGGGTGTTACCTTGAGTGTCTCTCTGTG‑T‑CC‑CTAGCTGTAACAGTCTCCTAACGTATCATCCTAAAGTCACCTCCTAACGTCCATCCTAAAGCCAAAACCTAAAGCCTACACCTAAAGACCCATCAAGTCAACGCCTATCTTAAAGTTACAACA‑‑‑‑‑‑‑‑‑‑‑‑‑taaagaccagacctaaagaacaaaact                                                                                                         >  1:146477/1‑296 (MQ=25)`  
                    `gtgtACGGACCTAAAGTT‑CCCCCATA‑GTGGGTACCTAAAGCCCAGCCAATCACCTAAAGTCAACCTTCGGTTGACCTTGAGGGTTCCCTAAGGGTTGGGGATGACCCTTGGGTTTGTCTTTGGGTGTTACCTTGAGTGTCTCTCTGTG‑T‑CC‑CTTTCTGTAACAGTCTCCTAAAGTATCCTCCTAAAGTCACCTCCTAACGTCCATCCTAAAGCCAACACCTAAAGCCTACACCTAAAGACCCATCAAGTCAACGCCTATCTTAAAGTTTAAACA‑‑‑‑‑‑‑‑‑‑‑‑‑Taaagaccagacctaaagaccagacct                                                                                                         >  1:269940/1‑301 (MQ=255)`  
                    `gtgtACGGACCTAAAGTT‑CCCCCATA‑GGGGGTACCTAAAGCCCAGCCAATCACCTAAAGTCAACCTTCGGTTGACCTTTAGGGTTCCCTAAGGGTTGGGGATGACCCTTGGGTTTGTCTTTGGGTGTTACCTTGAGTGTCTCTCTGTG‑T‑CC‑CTATCTGTAACAGTCTCCTAAAGTATCCTCCTAAAGTCACCTCCTAACGTCCATCCTAAAGCCAACACCTAAAGCCTACACCTAAAGACCCATCAAGTCAAAGCCTATCTTAAAGTTTAAACA‑‑‑‑‑‑‑‑‑‑‑‑‑taaagaccagacctaaagaccctgtct                                                                                                         >  1:197938/1‑295 (MQ=255)`  
                    `gtgtACGGACCTAAAGTT‑CCCCCATA‑GGGGGTACCTAAAGCCCAGCCAATCACCTAAAGTCAACCTTCGGTTGACCTTGAGGGTTCCCTAATGGTTGGGGATGACCCTTGGGTTTGTCTTTGGGTGTTACCTTGAGTGTCTCTCTGTG‑TCCC‑CTATCTGTTACAGTCTCCTAAAGTATCCTCCTAAAGTCACCTCCTAACGTCCATCCTAAAGCCAACACCTAAAGCCTACACCTAAAGACCCATCAAGTCAACGCCTATCTTAAAGTTTAAACA‑‑‑‑‑‑‑‑‑‑‑‑‑taaagaccagacctaaagaccagacc                                                                                                          >  1:239642/1‑301 (MQ=255)`  
                    `gtgtACGGACCTAAAGTT‑CCCCCATA‑GGGGGTACCTAAAGCCCAGCCAATCACCTAAAGTCAACCTTCGGTTGACCTTGAGGGTTCCCTAAGTGTTGGGGATGACCCTTTGGTTTGTCTTTGGGTGTTACCTTGAGTGTCTCTCTGTG‑T‑CC‑CTATCTGTTACAGTCTCCTAAAGTATCCTCCTAAAGTCACCTCCTAACGTCCATCCTAAAGCCAACACCTAAAGCCTACACCTAAAGACCCATCAAGTCAACGCCTATCTTAAAGTTTAAACA‑‑‑‑‑‑‑‑‑‑‑‑‑Taaagaccagacctaaagaccagacct                                                                                                         >  1:2070/1‑301 (MQ=255)`  
                    `gtgtACGGACCTAAAGTT‑CCCCCATA‑GGGGGTACCTAAAGCCCAGCCAATCACCTAAAGTCAACCTTCGGTTGACCTTGAGGGTTCCCTAAGGGTTGTGGATGACCCTTGGGTTTGTCTTTGGGTGTTACCTTGAGTGTCTCTCTGTG‑TCCC‑CTATCTGTTACAGTCTCCTAAAGTATCCTCCTAAAGTCACCTCCTAACGTCCATCCTAAAGCCAACACCTAAAGCCTACACCTAAAGACCCATCAAGTAAACGCCTATCTTAAAGTTTAAACA‑‑‑‑‑‑‑‑‑‑‑‑‑taaagacaaaacctaaagaccagacc                                                                                                          >  1:54726/1‑301 (MQ=255)`  
                    `gtgtACGGACCTAAAGTT‑CCCCCATA‑GGGGGTACCTAAAGCCCAGCCAATCACCTAAAGTCAACCTTCGGTTGACCTTGAGGGTTCCCTAAGGGTTGGGGATGACCCTTGGGTTTGTCTTTGTGTGTTACCTTGAGTGTCTCTCTGTG‑T‑CC‑C‑ATCTGTTACAGTCTCCTAAAGTATCCTCCTAAAGTCACCTCCTAACGTCCATCCTAAAGCCAACCCCTACAGCCTACACCTAAAGACCCATCAAGTCAACGCCTATCTTAAAGTTTAACCA‑‑‑‑‑‑‑‑‑‑‑‑‑TAaagaccagccctaaagaccagaccta                                                                                                        >  1:33630/1‑301 (MQ=255)`  
                    `gtgtACGGACCTAAAGTT‑CCCCCATA‑GGGGGTACCTAAAGCCCAGCCAATCACCTAAAGTCAACCTTCGGTTGACCTTGAGGGTTCCCTAAGGGTTGGGGATGACCCTTGGGTTTGTCTTTGGGTGTTACCTTGAGTGTCTCTCTGTGCC‑CC‑CTATATGTTACAGTCTCCTAAAGTATCCTCCTAAAGTCACCTCCTAACGTCCATCCTAAAGCCAACACCTAAAGCCTACACCTAAAGACCCATCAAGTCAACGCCTATCTTAAAGTTTAAACA‑‑‑‑‑‑‑‑‑‑‑‑‑taaagaccctgtctcttatacccatc                                                                                                          >  1:297538/1‑283 (MQ=255)`  
                    `gtgtACGGACCTAAAGTT‑CCCCCATA‑GGGGGTACCTAAAGCCCAGCCAATCACCTAAAGTCAACCTTCGGTTGACCTTGAGGGTTCCCTAAGGGTTGGGGATGACCCTTGGGTTTGTCTTTGGGTGTTACCTTGAGTGTCTCTCTGTG‑TCCC‑CTATCTGTAACAGTCTCCTAAAGTATCCTCCTAAAGTCACCTCCTAACGTCCATCCTAAAGCCAACACCTAAAGCCTACACCTAAAGACCCATCAAGTCAACGCCTATCTTAAAGTTTAAACA‑‑‑‑‑‑‑‑‑‑‑‑‑taaagaccaaacctaaagaccagacc                                                                                                          >  1:213176/1‑301 (MQ=255)`  
                    `gtgtACGGACCTAAAGTT‑CCCCCATA‑GGGGGTACCTAAAGCCCAGCCAATCACCTAAAGTCAACCTTCGGTTGACCTTGAGGGTTCCCTAAGGGTTGGGGATGACCCTTGGGTTTGTCTTTGGGTGTTACCTTGAGTGTCTCTCTGTG‑T‑CC‑CTATCTGTAACAGTCTCCTAAAGTATCCTCCTAAAGTCACCTCCTAACGTCCATCCTAAAGCCAACACCTACAGCCTACACATAAAGACCCATCAAATCAACGCCTATCTTAAAGTTTAAACA‑‑‑‑‑‑‑‑‑‑‑‑‑Taaagaccagacctaaagaccagacct                                                                                                         >  1:214986/1‑301 (MQ=255)`  
                    `gtgtACGGACCTAAAGTT‑CCCCCATA‑GGGGGTACCTAAAGCCCAGCCAATCACCTAAAGTCAACCTTCGGTTGACCTTGAGGGTTCCCTAAGGGTTGGGGATGACCCTTGGGTTTGTCTTTGGGTGTTACCTTGAGTGTCTCTCTGTG‑T‑CC‑CTATCTGTAACAGTCTCCTAAAGTATCCTCCTAAAGTCACCTCCTAACGTCCATCCTAAAGCCAACACC‑AAAGCCTACACCTAAAGACCCATCAAGTCAACGCCTATCTTAAAGTTTAAACA‑‑‑‑‑‑‑‑‑‑‑‑‑TAaagaccagacctaaagaccagaccta                                                                                                        >  1:204219/1‑301 (MQ=255)`  
                    `gtgtACGGACCTAAAGTT‑CCCCCATA‑GGGGGTACCTAAAGCCCAGCCAATCACCTAAAGTCAACCTTCGGTTGACCTTGAGGGTTCCCTAAGGGTTGGGGATGACCCTTGGGTTTGTCTTTGGGTGTTACCTTGAGTGTCTCTCTGTG‑T‑CC‑CTATCTGTAACAGTCTCCTAAAGTATCCTCCTAAAGTCACCTCCTAACGTACATCCTAAAGCCAACACCTAAAGCCTACACCTAAAGACCCATCAAGTCAACGCCTATCTTAAAGTTTAAACA‑‑‑‑‑‑‑‑‑‑‑‑‑Taaagaccagacctaaagaccagacct                                                                                                         >  1:263204/1‑301 (MQ=255)`  
                    `gtgtACGGACCTAAAGTT‑CCCCCATA‑GGGGGTACCTAAAGCCCAGCCAATCACCTAAAGTCAACCTTCGGTTGACCTTGAGGGTTCCCTAAGGGTTGGGGATGACCCTTGGGTTTGTCTTTGGGTGTTACCTTGAGTATCTCTCTGTG‑T‑CC‑CTATCTGTAACAGTCTCCTAAAGTATCCTCCTAAAGTCACCTCCTAACGTCCATCCTAAAGCCAACACCTAAAGCCTACACCTAAAGACCCATCAAGTCAACGCCTATCTTAAAGTTTAAACA‑‑‑‑‑‑‑‑‑‑‑‑‑Taaagaccagacctaaagaccagacct                                                                                                         >  1:143318/1‑301 (MQ=255)`  
                    `gtgtACGGACCTAAAGTT‑CCCCCATA‑AGGGGTACCTAAAGCCCAGCCAATCACCTAAAGTCAACCTTCGGTTGACCTTGAGGGTTCCCTAAGGGTTGTGGATGACCCTTGGGTTTGTCTTTGGGTGTTACCTTGAGTGTCTCTCTGTG‑T‑CC‑CTATCTGTAACAGTCTCCTAAAGTATCCTCCTAAAGTCACCTCCTAACGTCCATCCTAAAGCCAACACCTAAAGCCTACACCTAAAGACCCATCAAGTCAACGCCTATCTTAAAGTTCAAACA‑‑‑‑‑‑‑‑‑‑‑‑‑Taaagaccagacctaaagaccagacct                                                                                                         >  1:22047/1‑301 (MQ=255)`  
                    `gtgtACGGACCTAAAGTT‑CCCACATA‑GTGGGTACCTAAAGCCCAGCCAATCACCTAAACTCAACCTTCGGTTGACCTTGAGGGTTCCCTAAGGGTTGGGGATGACCCTTGGGTTTGTCTTTGGGTGTTACCTTGAGTGTCTCTCTGTG‑T‑CC‑CTATCTGTAACAGTCTCCTACAGTATCCTCCTAAAGCCACCTCCTAACGTCCATGCTAAAGCCAACACCTAAAGCCTACACCTAATGACCCATCCAGTCAACGCCTATCTTAACGTTTATACA‑‑‑‑‑‑‑‑‑‑‑‑‑Taaagcccagacctaaagaccagacct                                                                                                         >  1:159337/1‑301 (MQ=25)`  
                      `gtACGGACCTAAAGTT‑TCCCCATAGGGGGGTACCTAAAGCCCAGCCAATCACCTAAAGTCAACCTTCGGTTGACCTTGAGGGTTCCCTAAGGGTTGGGGATGACCCTTGGGTTTGTCTTTGGGTGTTACCTTGAGTGTCTCTCTGTG‑TCCC‑CTATCTGTTACAGTCTCCTAAAGTATCCTCCTAAAGTCACCTACTAACGTCCATCCTAAAGCCAACACCTAAAGCCTACACCTAAAGACCCATCAAGTCAACGCCTATCTTAAAGTTTAAACA‑‑‑‑‑‑‑‑‑‑‑‑‑Taaagaccagacctaaagaccagacct                                                                                                         >  1:31971/1‑301 (MQ=255)`  
                      `gtACGGACCTAAAGTT‑CCCCCATA‑GGGGGTACCTAAAGCCCAGCCAATCACCTAAAGTCAACCTTCGGTTGACCTTGAGGGTTCCCTAAGGGTTGGGGATGACCCTTGGGTTTGTCTTTGGGTGTTACCTTGAGTGTCTCTCTGTG‑TCCC‑CTATCTGTAACAGTCTCCTAAAGTATCCTCCTAAAGTCACCTCCTAACGTCCATCCTAAAGCCAACACCTAAAGCCTACACCTAAAGACCAATCAAGTCAACGCCTATCTTAAAGTTTAAACA‑‑‑‑‑‑‑‑‑‑‑‑‑TAaagacaagacctaaagaccagaccta                                                                                                        >  1:39837/1‑301 (MQ=255)`  
                      `gtACGGACCTAAAGTT‑CCCCCATA‑GGGGGTACCTAAAGCCCAGCCAATCACCTAAAGTCAACCTTCGGTTGACCTTGAGGGTTCCCTAAGGGTTGGGGATGACCCTTGGGTTTGTCTTTGGGTGTTACCTTGAGTGTCTCTCTGTG‑T‑CC‑CTATCTG‑TACAGTCTCCTAAAGTATCCTCCTAAAGTCACCTCCTAACGTCCATCCTAAAGCCAACACCTAAAGCCTACACCTAAAGACCCATCAAGTCAACGCCTATCTTAAAGTTTAAACA‑‑‑‑‑‑‑‑‑‑‑‑‑TAAAgaccagacctaaagaccagacctaaa                                                                                                      >  1:164552/1‑301 (MQ=255)`  
                      `gtACGGACCTAAAGTT‑CCCCCATA‑GGGGGTACCTAAAGCCCAGCCAATCACCTAAAGTCAACCTTCGGTTGACCTTGAGGGTTCCCTAAGGGTTGGGGATGACCCTTGGGTTTGTCTTTGGGTGTTACCTTGAGTGTCTCTCTGTG‑T‑CC‑C‑ATCTGTTACAGTCTCCTAAAGTATCCTCCTAAAGTCACCTCCTAACGTCCATCCTAAAGCCAACACCTAAAGCCTACACCTAAAGACCCATCAAGTCAACGCCTATCTTAAAGTTTAAACA‑‑‑‑‑‑‑‑‑‑‑‑‑TAAAgaccagacctaaagaccagacctaaa                                                                                                      >  1:126497/1‑301 (MQ=255)`  
                      `gtACGGACCTAAAGTT‑CCCCCATA‑GGGGGTACCTAAAGCCCAGCCAATCACCTAAAGTCAACCTTCGGTTGACCTTGAGGGTTCCCTAAGGGTTGGGGATGACCCTTGGGTTTGTCTTTGGGTGTTACCTTGAGTGTCTCTCTGTG‑T‑CC‑C‑ATCTGTTACAGTCTCCTAAAGTATCCACCTAAAGTCACCACCTAACGTCCATCCTAAAGACAACACCTAAAGCACACACCTAAAGCCACATCAAGTCAAAGCCCATCTTAAAGTTTAAACA‑‑‑‑‑‑‑‑‑‑‑‑‑taaagacaagaccgaaagctgtatcttata                                                                                                      >  1:326798/1‑289 (MQ=21)`  
                      `gtACGGACCTAAAGTT‑CCCCCATA‑GGGGGTACCTAAAGCCCAGCCAATCACCTAAAGTCAACCTTCGGTTGACCTTGAGGGTTCCCTAAGGGTTGGGGATGACCCTTGGGTTTGTCTTTGGGTGTTACCTTGAGTGTATCTCTGTG‑TCCC‑CTATCTGTTACAGTCTCCTAAAGTTTCCTCCTAAAGTCACCTCCTAACGTCCATCCTAAAGCCAACACCTAAAGCCTACACCTAAAGACCCATCAAGTCAACGCCTATCTTAAAGTTTAAACA‑‑‑‑‑‑‑‑‑‑‑‑‑TAaagaccagacctaaagaccagaccta                                                                                                        >  1:341678/1‑301 (MQ=255)`  
                      `gtACGGACCTAAAGTT‑CCCCCATA‑‑GGGGTACCTAAAGCCCAGCCAATCACCTAAAGTCAACCTTCGGTTGACCTTGAGGGTTCCCTAAGGGTTGGGGATGACCCTTGGGTTTGTCTTTGGGTGTTACCTTGAGTGTCTCTCTGTG‑T‑CC‑CTATCTGTAACAGTCTCCTAAAGTATCCTCCTAAAGTCACCTCCTAACGTCCATCCTAAAGCCAACACCTAAAGCCTACACCTAAAGACCCATCAAGTCAACGCCTATCTTAAAGTTTAAACA‑‑‑‑‑‑‑‑‑‑‑‑‑taaagaccagacctaaagctgtctcttata                                                                                                      >  1:231138/1‑289 (MQ=255)`  
                        `caggACCTAAAGTT‑CCCCCATA‑GGGGGTACCTAAAGCCCAGCCAATCACCTAAAGTCAACCTTCGGTGTACCTTGAGGGTTCCCTAAGGGTTGGGGATGACCCTTGGGTTTGTCTTTGGGTGTTACCTTGAGTGTCTCTCGGTG‑T‑CC‑CTATCTGTTACAGTCTACTAAAGTATCCTCCTAAAGTCACCTCCTAACGTCCATCCTAAAGCCAACACCTAAAGCCTACACCTAAAGACCCATCAAGTCAACGCCTATCTTAAAGTTTAAACA‑‑‑‑‑‑‑‑‑‑‑‑‑TAAAGaccagacctaaataccagacctaaag                                                                                                     <  1:72605/299‑1 (MQ=255)`  
                           `gACCTAAAGTTCCCCCCATA‑GGGTGTACCTAAAGCCCAGCCAATCACCTAAAGTCAACCTTCGGTTGACCTTGAGGGTTCCCTAAGGGTTGGGGATGACCCTTGGGTTTGTCTTTGGGTGTTACCTTGAGTGTCTCTCTGTG‑T‑CC‑CTATCTG‑TACAGTCTCCTAAAGTATCCTCCTAAAGTCACCTCCTAACGTCCATCCTAAAGCCAACACCTAAAGCCTACACATAAAGACCTATCAAGTCAACGCCTATCTTAAAGTTTAAACA‑‑‑‑‑‑‑‑‑‑‑‑‑TAAAGACCAGACATAAAGACCAGACCTAAAGaca                                                                                                  >  1:117644/1‑301 (MQ=255)`  
                           `gACCTAAAGTT‑CCCCCATA‑GTGGGTACCTAAAGCCCAGCCAATCACCTAAATTCAACCTTCGGTTGACCTTGAGGGTTCCCTAAGGGTTGGGGATGACCCTTGGGTTTGTCTTTGGGTGTTACCTTGAGTGTCTCTCTTTG‑T‑CC‑CTATCTGTAACAGTCTCCTACAGTATCCTCCTAAAGTCACCTCCTAACGTCCATCATAAATCCAACACCTAAAGCCTACACCTAAAGACCCATCAAGTCAACGCCTATCTTAAAGTTTAAACA‑‑‑‑‑‑‑‑‑‑‑‑‑TAAAGACCAGACCTAAAGACCAGACCTAACGaca                                                                                                  >  1:128696/1‑301 (MQ=255)`  
                           `gACCTAAAGTT‑CCCCCATA‑GGGGGTACCTAAAGCCCAGCCAATCACCTAAAGTCAACCTTCGGTTGACCTTGAGGGTTCCCTAAGGGTTGGGGATGACCCTTTGGTTTGTCTTTGGGTGTTACCTTGAGTGTCTCTCTGTG‑T‑CC‑C‑ATCTGTTACAGTCTCCTAAAGTATCCTCCTAAAGTCACCTCCTAACGTCCATCCTAAAGCCAACACCTCAAGCCTACACCTAAAGACCCATCAAGTCAACGCCTATCTTAAAGTTTAAACA‑‑‑‑‑‑‑‑‑‑‑‑‑taaagaccagacctaaagctgtcccttatacacat                                                                                                 >  1:68989/1‑284 (MQ=255)`  
                           `gACCTAAAGTT‑CCCCCATA‑GGGGGTACCTAAAGCCCAGCCAATCACCTAAAGTCAACCTTCGGTTGACCTTGAGGGTTCCCTAAGGGTTGGGGATGACCCTTGGGTTTGTCTTTGGGTGTTACCTTGAGTGTCTCTCTGTG‑TCCC‑CTATCTGTTACAGTCTCCTAAAGTATCCTCCTAAAGTCACTTCCTACCGTCCATCCTAAAGCCAACACCTAAAGCCTACACCTAAAGACCCATCAAGTCAACGCCTATCTTAAAGTTTAAACA‑‑‑‑‑‑‑‑‑‑‑‑‑TAAAGAccagcactaaagaccagacctaaagaa                                                                                                   >  1:153092/1‑300 (MQ=255)`  
                           `gACCTAAAGTT‑CCCCCATA‑GGGGGTACCTAAAGCCCAGCCAATCACCTAAAGTCAACCTTCGGTTGACCTTGAGGGTTCCCTAAGGGTTGGGGATGACCCTTGGGTTTGTCTTTGGGTGTTACCTTGAGTGTCTCTCTGTG‑T‑CC‑CTATCTGTAACAGTCTCCTAAAGTATCCTCCTAAAGTCACCTCCTAACGTCCATCCTAAAGCCAACACCTAAAGCCTACACCTAAAGACCCATCAAGTCAACGCCTATCTTAAAGTTTAAACA‑‑‑‑‑‑‑‑‑‑‑‑‑taaagaccagacctaaagacctgtctcttataca                                                                                                  >  1:113179/1‑288 (MQ=255)`  
                           `gACCTAAAGTT‑CCCCCATA‑GGGGGTACCTAAAGCCCAGCCAATCACCTAAAGTCAACCTTCGGTTGACCTTGAGGGTTCCCTAAGGGTTGGGGATGACCCTTGGGTTTGTCTTTGGGTGTTACCTTGAGTGTCTCTCTGTG‑T‑CC‑C‑ATCTGTTACAGTCTCCTAAAGTATCCTCCTAAAGTCACCTCCTACCGTCCATCCTAAAGCCAACACCTAAAGCCTACACCTAAAGACCCATCAAGTCAACGCCTATCTTAAAGTTTAAACA‑‑‑‑‑‑‑‑‑‑‑‑‑TAAAGACCAGACCTAAAGACCAGACCTAAATacac                                                                                                 >  1:265369/1‑301 (MQ=255)`  
                           `gACCTAAAGTT‑CCCCCATA‑GGGGGTACCTAAAGCCCAGCCAATCACCTAAAGTCAACCTTCGGTTGACCTTGAGGGTTCCCTAAGGGTTGGGGATGACCCTTGGGTTTGTCTTTGGGTGTTACCTTGAGTGTCTCTCTGTG‑T‑CC‑C‑ATCTGTTACAGTCTCCTAAAGTATCCTCCTAAAGTCACCTCCTAACGTCCATCCTAAAGCCAACACCTAAAGCCTACACCTAAAGACCCATCAAGTCAACGCCTATCTTAAAGTTTAAACA‑‑‑‑‑‑‑‑‑‑‑‑‑TAAAGACCAGACCTAAAGACCAGACCTAAAGacac                                                                                                 >  1:254978/1‑301 (MQ=255)`  
                           `gACCTAAAGTT‑CCCCCATA‑GGGGGTACCTAAAGCCCAGCCAATCACCTAAAGTCAACCTTCGGTTGACCTTGAGGGTTCCCTAAGGGTTGGGGATGACCCTTGGGTTTGTCTTTGGGTGTTACCTTGAGTGTCTCTCTGTG‑T‑CC‑C‑ATCTGTTACAGTCTCCTAAAGTATCCTCCTAAAGTCACCTCATAACGTCAATCCTAAAGCCAACACCTAAAGCCTACACCTAAAGACCCATCAAGTCAACGCCTATCTTAAAGTTTAAACA‑‑‑‑‑‑‑‑‑‑‑‑‑TAAAGCCCAGACCTAAAGACCAGACCTAAAGacac                                                                                                 >  1:123111/1‑301 (MQ=255)`  
                             `tataagagacagcccATA‑GGGGGTACCTAAAGCCCAGCCAATCACCTAAAGTCAACCTTCGGTTGACCTTGAGGGTTCCCTAAGGGTTGGGGATGACCCTTGGGTTTGTCTTTGGGTGTTACCTTGAGTGTCTCTCTGTG‑T‑CC‑CTATCTGTAACAGTCTCCTAAAGTATCCTCCTAAAGTCACCTCCTAACGTCCATCCTAAAGCCAACACCTAAAGCCTACACCTAAAGACCCATCAAGTCAACGCCTATCTTAAAGTTTAAACA‑‑‑‑‑‑‑‑‑‑‑‑‑TAAAGACCAGACCTAAAGACCAGACCTAAAGacac                                                                                                 <  1:287500/289‑1 (MQ=255)`  
                             `acTAAAGTT‑CCCCCATA‑GGGGGTACCTAAAGCCCAGCCAATCACCTAAAGTCAACCTCCGGTTGACCTTGAGGGTTCCCTAAGGGTTGTGGATTACCCTTGGGTTTGTCTATGGGTGTTACCTTGAGTGTCTCTCTGTG‑TCCC‑CTATCTGTTACAGTCTCCTAAAGTATCCTCCTAAAGTCACCTCCTAACGTCCATCCTAAAGCCAACACCTAAAGCCTACACCTAAAGACCCATCAAGTCAACGCCTATCTTAAAGTTTAAACA‑‑‑‑‑‑‑‑‑‑‑‑‑TAAAGACCAGACCTAAAGACCAGACCTAAAGacac                                                                                                 <  1:50702/300‑1 (MQ=255)`  
                                `aaaGTT‑CCCCCATA‑GGGGGTACCTAAAGCCCAGCCAATCACCTAAAGTCAACCTTCGGTTGACCTTGAGGGTTCCCTAAGGGTTGGGGATGACCCTTGGGTTTGTCTTTGGGTGTTACCTTGAGTGTCTCTCTGTG‑T‑CC‑CTATCTGTAACAGTCTCCTAAAGTATCCTCCTAAAGTCACCTCCTAACGTCCATCCTAAAGCCAACACCTAACGCCTACACCTAAAGACCCATCAGGTCAACGCCTATCTTAAAGTTTAAACA‑‑‑‑‑‑‑‑‑‑‑‑‑taaagaccagacctcaagacgccacgtagtgacactaaa                                                                                             >  1:174237/1‑282 (MQ=25)`  
                                     `t‑CCCCCATA‑GGGGGTACCTAAAGCCCAGCCAATCACCTACAGTCAACCTTCGGTTGACCTTGATGGTTCCCTACGGGTTGGGGATTACCCTTGGGTTTGTCTTTGTGTGTTACCTTGATTGTCTCTCTGTT‑T‑CC‑CTATCTGTTCCAGTCTCCTTACTTATCCTCCTACAGTCACCTCCTAACGTCCATCCTAACTCCAACACCTAAAGCCTACACCTAAAGACCCATCACGTCAACGCCTATCTTAAAGTTTAACCA‑‑‑‑‑‑‑‑‑‑‑‑‑TAAAGACCAGACCTAAAGACCAGACCTAACGACACTAcctcaaa                                                                                        >  1:209357/1‑295 (MQ=25)`  
                                      `tgtgtataagagacagaCCCAAAGCCCATCCAATCACCTAAAGTCAACCTTCGGTTGACCTTGAGGGTTCCCTAATGGTTGGGGATGACCCTTGGGTTTGTCTTTGGGTGTTACCTTTAGTGTCTCTCTGTG‑T‑CC‑CTATCTGTAACAGTCTCCTAAAGTATCCTCCTAAAGTCACCTCCTAACGTCCATCCTAAAGCCAACACCTAAAGCCTACACCTAAAGACCCATCAAGTCAACGCCTATCTTAAAGTTTAAACA‑‑‑‑‑‑‑‑‑‑‑‑‑TAAAGACCAGACCTAAAGACCAGACCTAAAGACACTACATaaa                                                                                         <  1:334095/285‑1 (MQ=37)`  
                                       `cccccATA‑GGGGGTACCTAAAGCCCAGCCAATCACCTAAAGTCAACCTTCGGTTGACCTTGAGGGTTCCCTAAGGGTTGGGGATGACCCTTGGGTTTGTCTTTGGGTGTTACCTTGAGTGTCTCTCTGTG‑T‑CC‑CTATCTGAAACAGTCTCCTAAAGTATCCTCCTAAAGTCACCTCCTAACGTCCATCCTAAAGCCAACACCTAAAGCCTACACCTAAAGACCCATCAAGTCAACGCCTATCTTAAAGTTTAAACA‑‑‑‑‑‑‑‑‑‑‑‑‑TAAAGACCAGACCTAAAGACCAGACCTAAAGACCCTACATAAaga                                                                                       >  1:304125/1‑301 (MQ=255)`  
                                       `ccagcctA‑GTGGGTACCGAAGTCCCAGCCAATCACCTAAAGTCAACCTTCGGTTGTCCTTGATGGTTCCCTAAGGGTTGGTGATTACCCTTGTGTTTGTCTTTGGGTGTTACCTTGAGTGTCTCTCTGTG‑T‑CC‑CTATCTGTAACAGTCTCCTAAAGTATCCTCCTAAAGTCACCTCCTAACGTCCATCCTAATGCCAACACCTAAAGCCTACACCTAAAGACCCATAAAGTCAACGCCTATCTTAAAGTTTAAACA‑‑‑‑‑‑‑‑‑‑‑‑‑TAAAGACCAGACCTAAAGACCAGACCTAAAGACACTACATAAaga                                                                                       <  1:3646/295‑1 (MQ=37)`  
                                        `ccccATA‑GGGGGTACCTAAAGCCCAGCCAATCACCTAAAGTCAACCTTCGGTTGACCTTGAGGGTTCCCTAAGGGTTGGGGATGACCCTTGGGTTTGTCTTTGGGTGTTACCTTGAGTGTCTCTCTGTG‑T‑CC‑CTATCTGTAACAGTCTCCTAAAGTATCCTCCTAAAGTCACCTCCTAACGTCCATCCTAAAGCCAACACCTAAAGCCTACACCTAAAGACCCATCAAGTCAACGCCTATCTTAAAGTTTAAACA‑‑‑‑‑‑‑‑‑‑‑‑‑TAAAGACCAGACCTAAAGACCAGACCTAAAGACACTACATAAagac                                                                                      <  1:103402/301‑1 (MQ=255)`  
                                        `aataagagacaggTACCTAAAGCCCAGCCAATCACCTAAAGTCAACCTTCGGTTGACCGTGAGGGTTCCCTAATGGTTGGGGATGACCCTTGGGTGTGTCTTTGGGTGTTACCTTGAGTGTCTCTCTGTG‑T‑CC‑ATAACTG‑TACAGTCACCTAAAGTATCCTCCGAAAGTCACCTCCTAACGTCAATCCGAAAGCCAACACCTAAAGCCGACACATAAAGACCCATGAAGGCAAAGCCTATCGTAAAGTGTAAAAA‑‑‑‑‑‑‑‑‑‑‑‑‑TAAAGACAAGACCTAAAGAACAGACCTAAAGACACTACATAAagac                                                                                      <  1:237496/290‑1 (MQ=21)`  
                                         `cccATA‑GGGTGTACCTAAAGCCCAGCCAATCACCTAAAGTCAACCTTCGGTTGACCTTGAGGGTTCCCTAAGGGTTGGGGATGACCCTTGGGTTTGTCTTTGGGTGTTACCTTGAGTGTCTCTCTGTG‑T‑CC‑CTATCTG‑TACAGTCTCCTAAAGTATCCTCCTAAAGTCACCTCCTAACGTCCATCCTAAAGCCAACACCTAAAGCCTACACCTAAAGACCCATCAAGTCAACTCCTATCTTAAAGTTTAAACA‑‑‑‑‑‑‑‑‑‑‑‑‑TAAAGACCAGACCTAAAGACCAGACCTAAAGACACTACATAAagctgt                                                                                    >  1:192698/1‑297 (MQ=255)`  
                                         `cccATA‑GGGGGTACCTAAAGCCCAGCCAATCACCTAAAGTCAACCTTCGGTTGACCTTGAGGGTTCCCTAAGGGTTGGGGATGACCCTTGGGTTTGTCTTTGGGTGTTACCTTGAGTGTCTCTCTGTG‑TCCC‑CTATCTGTTACAGTCTCCTAAAGTATCCTCCTAAAGTCACCTCATAACGTCCATCCTAAAGCCAACACCTAAAGCCTACACCTAAAGACCCATCAAGTCAACGCCTATCTTAAAGTTTAAACA‑‑‑‑‑‑‑‑‑‑‑‑‑TAAAGACCAGACCTAAAGACCAGACCTAAAGACACTACATAAagac                                                                                      <  1:176381/301‑1 (MQ=255)`  
                                         `cccATA‑GGGGGTACCTAAAGCCCAGCCAATCACCTAAAGTCAACCTTCGGTTGACCTTGAGGGTTCCCTAAGGGTTGGGGATGACCCTTGGGTTTGTCTTTGGGTGTTACCTTGAGTGTCTCTCTGTG‑T‑CC‑CTATCTGTTACAGTCTCCTAAAGTATCCTCCTAAAGTCACCTCCTAACGTCCATCCTAAAGCCAACACCTAAAGCCTACACCTAAAGACCCATCAAGTCAACGCCTATCTTAAAGTTTAAACA‑‑‑‑‑‑‑‑‑‑‑‑‑TAAAGaccagacctaaagaccagacctaaagctgtctcttatacaca                                                                                     >  1:120588/1‑285 (MQ=255)`  
                                         `cccATA‑GGGGGTACCTAAAGCCCAGCCAATCACCTAAAGTCAACCTTCGGTTGACCTTGAGGGTTCCCTAAGGGTTGGGGATGACCCTTGGGTTTGTCTTTGGGTGTTACCTTGAGTGTCTCTCTGTG‑T‑CC‑CTATCTGTAACAGTCTCCTAAAGTATCCTCCTAAAGTCACCTCCTAACGTCCATCCTAAAGCCAACACCTAAAGCCTACACCTAAAGACCCATCAAGTCAACGCCTATCTTAAAGTTTAAACA‑‑‑‑‑‑‑‑‑‑‑‑‑TAAAGACCAGACCTAAAGACCAGACCTAAAGACACTACATAAagctg                                                                                     >  1:57004/1‑298 (MQ=255)`  
                                         `cccATA‑GGGGGTACCTAAAGCCCAGCCAATCACCTAAAGTCAACCTTCGGTTGACCTTGAGGGTTCCCTAAGGGTTGGGGATGACCCTTGGGTTTGTCTTTGGGTGTTACCTTGAGTGTCTCTCTGTG‑T‑CC‑CTATCTGTAACAGTCTACTAAAGTATCCTACTAAAGTCACCTCCTAACGTCCATCCTAAAGCCAACACCTAAAGCCTACACCTAAAGACCCATCAAGTCAACGCCTATCTTAAAGTTTAAACA‑‑‑‑‑‑‑‑‑‑‑‑‑TAAAGACAAGACCTAAAGACCAGACCTAAAGACAATACATAAagctg                                                                                     >  1:8756/1‑298 (MQ=255)`  
                                         `cccACA‑TGGGGTACCTAAAGCCCAGCCAATCACCTAAAGTCAACCTTCGGTTGACCTTGAGGGTTCCCTAAGGGTTGGGGATGACCCTTGGGTTTGTCTTTGGGTGTTACCTTGAGTGTCTCTCTGTG‑T‑CC‑CTATCTGTAACAGTCTCCTAAAGTATCCTCCTAAAGTCACCTCCTAACGTCCATCCTAAAGCCAACACCTAAAGCCTACACCTAAAGACCCATCACGTCAACGCCTATCTTAAAGTTTAAACA‑‑‑‑‑‑‑‑‑‑‑‑‑TAAAGACCAGACCTAAAGACCAGACCTAAAGACACTACATAAagacc                                                                                     >  1:96995/1‑301 (MQ=255)`  
                                              `ggctcggagatgtgtataagagacagcACCTAAAGTCAACCTTCGGTTGACCTTGAGGGTTCCCTAAGGGTTGGGGATGACCCTTGGGTTTGTCTTTGGGTGTTACCTTGAGTGTCTCTCTGTG‑T‑CC‑C‑ATCTGTTACAGTCTCGTAAAGTATCCTCCTAAAGTCACCTCCTAACGTCCATCCTAAAGCCAACACCTAAAGCCTACACCTAAAGACCCATCAAGTCAACGCCTATCTTAAAGTTTAAACA‑‑‑‑‑‑‑‑‑‑‑‑‑TAAAGACCAGACCTAAAGACCAGACCTAAAGACACTACATAAagaccagacc                                                                                <  1:252137/275‑1 (MQ=38)`  
                                                `gggggTACCTAAAGCCCAGCCAATCACCTAAAGTCAACCTTCGGTTGACCTTTAGGGTTCCCTAAGTGTTGGGGATGACCCTTGGGTTTGTCTTTGGGTGTTACCTTGAGTGTCTCTCTGTG‑T‑CC‑CTATCTGTAACAGTCTCCTAAAGTATCCTCCTAAAGTCACCTCCTAACGTCCATCCTAAAGCCAACACCTAAAGCCTACACCTAAAGACCCATCAAGTCAACGCCTATCTTAAAGTTTAAACA‑‑‑‑‑‑‑‑‑‑‑‑‑TAAAGACcagacctaaagaccagacctaaagacctgtctcttatacacatctc                                                                               >  1:322332/1‑281 (MQ=255)`  
                                                `gggggTACCTAAAGCCCAGCCAATCACCTAAAGTCAACCTTCGGTTGACCTTGAGGGTTCCCTAATGGTTTGGGATGACCCTTGGGTTTGTCTTTGGGTGTTACCTTGAGTGTCTCTCTGTG‑TCCC‑CTATCTGTTACAGTCTCCTAAAGTATCCTCCTAAAGTCACCTCCTAACGTCCATCCTAAAGCCAACACCTAAAGCCTACACCTAAAGACCCATCAAGTCAACGCCTTTCTTAAAGTTTAAACA‑‑‑‑‑‑‑‑‑‑‑‑‑TAAAGACCAGACCTAAAGACCAGACCTAAAGACACTACATAAagaccagaca                                                                                >  1:257609/1‑300 (MQ=255)`  
                                                `gggggTACCTAAAGCCCAGCCAATCACCTAAAGTCAACCTTCGGTTGACCTTGAGGGTTCCCTAAGGGTTGGGGATGACCCTTGGGTTTGTCTTT‑GGTGTTACCTTGAGTGTCTCTCTGTG‑TCCC‑CTATCTGTTACAGTCTCCTAAAGTATCCTCCTAAAGTCACCTCCTAACGTCCATCCTAAAGCCAACACCTAAAGCCTACACCTAAAGACCCATCAAGTCAACGCCTATCTTAAAGTTTAAACA‑‑‑‑‑‑‑‑‑‑‑‑‑TAAAGACCAGACCTAAAGACCAGACCTAAAGACACTACATAAAGACCAGACCt                                                                               >  1:296032/1‑301 (MQ=255)`  
                                                 `ggggTACCTAAAGCCCAGCCAATCACCTAAAGTCAACCTTCGGTTGACCTTGAGGGTTCCCTAAGGGTTGGGGATGACCCTTGGGTTTGTCTTTGGGTGTTACCTTGAGTGTCTCTCTGTG‑T‑CC‑CTATCTGTAACAGTCTCCTAAAGTATCCTCCTAAAGTCACATCCTAACGTCCATCCTAAAGCCAACACCTAAAGCCTACACCTAAAGACCCATCAAGTCAACGCCTATCTTAAAGTTTAAACA‑‑‑‑‑‑‑‑‑‑‑‑‑TAAAGACCAGACCTAAAGACCAGACCTAAAGACACTACATAAAGACCAGACCTa                                                                              >  1:283022/1‑301 (MQ=255)`  
                                                      `aCCTAAAGCCCAGCCAATCACCTAAAGTCCACCTTCGGTTGACCTTGAGGGTTCCCTAAGGGTTGGGGATGACCCTTGGGTTTGTCTTTGGGTGTTACCTTGAGTGTCTCTCTGTG‑TCCC‑CTATCTGTTACAGTCTCCTAAAGTATCCTCCTAAAGTCACCTCCTAACGTCCATCCTAAAGCCAACACCTAAAGCCTACACCTAAAGACCCATCAAGTCAACGCCTATCTTAAAGTTTAAACA‑‑‑‑‑‑‑‑‑‑‑‑‑TAAAGACCAGACCTAAAGACCAGACCTAAAGACCAGACCTAAAGACACTACATAAAGa                                                                          <  1:306159/301‑1 (MQ=37)`  
                                                      `aCCTAAAGCCCAGCCAATCACCTAAAGTCAACCTTCGGTTGACCTTGAGGGTTCCCTAATGGTTGGGGATGAACCTTGGGTTTGTCTTTGGGTGTTACCTTGAGTGTCTCTCTGTG‑T‑CC‑C‑ATCTGTTACAGTCTCCTAAAGTATCCTCCTAAAGTCACCTCCTAACGTCCATCCTAAAGCCAACACCTAAAGCCTACACCTAAAGACCCATCAAGTCAACGCCGATCTTAAAGTTTAAACA‑‑‑‑‑‑‑‑‑‑‑‑‑TAAAGACCAGACCTAAAGACCAGACCTAAAGACACTACATAAAGACCAGACCTAAAGACg                                                                        >  1:312986/1‑301 (MQ=255)`  
                                                      `aCCTAAAGCCCAGCCAATCACCTAAAGTCAACCTTCGGTTGACCTTGAGGGTTCCCTAAGGGTTGGGGATGACCCTTGGGTTTGTCTTTGGGTGTTACCTTGAGTGTCTCTCTGTG‑T‑CC‑CTATCTG‑TACAGTCTCCTAAAGTATCCTCCTAAAGTCACCTCCTAACGTCCATCCTAAAGCCAACACCTAAAGCCTACACCTAAAGACCCATCAAGTCAACGCCTATCTTAAAGTTTAAACA‑‑‑‑‑‑‑‑‑‑‑‑‑TAAAGACCAGACCTAAAGACCAGACCTAAAGACACTACATAAAGACCAGACCTAAAGACg                                                                        >  1:243176/1‑301 (MQ=255)`  
                                                       `ccTAAAGCCCAGCCAATCACCTAAAGTCAACCTTCGGTTGACCTTGAGGGTTCCCTAAGGGTTTGGGATGACCCTTGGGTTTGTCTTTGGGTGTTACCTTGAGTGTCTCTCTGTG‑T‑CC‑CTATCTGTAACAGTCTCCTAAAGTATCCTCCTAAAGTCACCTCCTAACGTCCATCCTAAAGCCAACACCTAAAGCCTACACCTAAAGACCCATCAAGTCAACGCCTATCTTAAAGTTTAAACA‑‑‑‑‑‑‑‑‑‑‑‑‑TAAAGACCAGACCTAAAGACCAGACCTAAAGACACTACATAAAGACCAGACCtgtctctt                                                                        >  1:89926/1‑294 (MQ=255)`  
                                                       `ccTAAAGCCCAGCCAATCACCTAAAGTCAACCTTCGGTTGACCTTGAGGGTTCCCTAAGGGTTGGGGATGACCCTTGGGTTTGTCTTTGGGTGTTACCTTGAGTGTCTCTCTGTG‑T‑CC‑CTATCTGTAACAGTCTCCTAAAGTATCCTCCTAAAGTCACCTCCTAACGTCCATCCTAAAGCCAACACCTAAATCCTACACCTAAAGACCCATCAAGTCAACGCCTATCTTAAAGTTTAAACA‑‑‑‑‑‑‑‑‑‑‑‑‑TAAAGACCAGACCTAAAGACCAGACCTAAAGACACTACATAAagaccctgtctcttatac                                                                        >  1:9524/1‑288 (MQ=255)`  
                                                                  `gCCAATCACCTAAAGTCAACCTTCGGTTGACCTTGAGGGTTCCCTAAGGGTTGGGGATGACCCTTGGGTTTGTCTTTGGGTGTTACCTTGAGTGTCTCTCTGTG‑T‑CC‑CTATCTGTAACAGTCTCCTAAAGTATCCTCCTAAAGTCACCTCCTAACGTCCATCCTAAAGCCAACACCTAAAGCCTACACCTAAAGACCCATCAAGTCAACGCCTATCTTAAAGTTTAAACA‑‑‑‑‑‑‑‑‑‑‑‑‑TAAAGACCAGACCTAAAGACCAGACCTAAAGACACTACATAAAGACCAGACCTAAAGACGCCTTGTTGTTa                                                             >  1:232969/1‑301 (MQ=255)`  
                                                                    `cAATCACCTAAAGTCAACCGTCGGTTGACCTTTAGGGTTCCCTAAGGGTTGGGGATGACCCTTGGGTTTGTCTTTGGGTGTTACCTTGAGTGTCTCTCTGTG‑T‑CC‑CTATCTGTAACAGTCTCCTAAAGTATCCTCCTAAAGTCACCTCCTAACGTCCATCCTAAAGCCAACACCTAAAGCCTACACCTAAAGACCCATCAAGTCAACGCCTATCTTAAAGTTTAAACA‑‑‑‑‑‑‑‑‑‑‑‑‑TAAAGACCAGACCTAAAGACCAGACCTAAAGACACTACATAAAGACCAGACCTAAAGACGCCTTGTTGTTAGc                                                           <  1:179314/301‑1 (MQ=255)`  
                                                                        `cACCTAAAGTCAACCTTCGGTTGACCTTGAGGGTTCCCTAAGGGTTGGGGATGACCCTTGGGTTTGTCTTTGGGTGTTACCTTGAGTGTCTCTCTGTG‑T‑CCACTATCTGTTACAGTCTCCTAAAGTATCCTCCTAAAGTCACCTCCTAACATCCATCCTAAAGCCAACACCTAAAGCCTACACCTCAAGACCCATCAAGTCAACGCCTATCTTAAAGTTTAAACA‑‑‑‑‑‑‑‑‑‑‑‑‑TAAAGACCAGACCTAAAGACCAGACCTAAATACACTACATAAAGACCAGACCTAAAGACGCCTTGTTGTTAGCAAt                                                        >  1:293308/1‑301 (MQ=255)`  
                                                                             `aaaGTCAACCTTCGGTTGACCTTGAGGGTTCCCTAAGGGTTGGGGATGACCCTTGGGTTTGTCTTTGGGTGTTACCTTGAGTGTCTCTCTGTG‑T‑CC‑CTATCTG‑TACAGTCTCCTAAAGTATCCTCCTAAAGTCACCTCCTAACGTCCATCCTAAAGCCAACACCTAAAGCCTACACCTAAAGACCCATCAAGTCAACGCCTATCTTAAAGTTTAAACA‑‑‑‑‑‑‑‑‑‑‑‑‑TAAAGACCAGACCTAAAGACCACATCTACAGACACAACATAAAGACCAGACCTAAAGACGCCATGTTGTTAGCCACAAAGTGa                                                 >  1:315660/1‑301 (MQ=255)`  
                                                                                   `aaCCTTCGGTTGACCTTGGGGGTTCCCTAAGGGTTGGGGATGACCCTTGGGTTTGTCGTTGGGTGTTACCTTGAGTGTCTCTCTGTG‑T‑CC‑CTATCTGTTACATTCTCCTAAAGTATCCTCCTAAAGTCACCTCCTAACGTCCATCCTAAAGCCAACACCTAAAGCCTACACCTAAAGACCCAACAAGTCAACGCCTATCTTAAAGTTTAAACA‑‑‑‑‑‑‑‑‑‑‑‑‑TAAAGACCAGACCTAAAGACCAGACCTAAAGACACTACATAAAGACCAGACCTAAAGACGCCTTGTTGTTAGCCATAAAGTGATAAcc                                            <  1:223743/301‑1 (MQ=255)`  
                                                                                   `aaCCTTCGGTTGACCTTGAGGGTTCCCTAAGGGTTTGGGATGACCCTTGGGTTTGTCTTTGGGTGTTACCTTGAGTGTCTCTCTGTG‑T‑CC‑CTATCTGTAACAGTCTCCTAAAGTATCCTCCTAAAGTCACCTCCGAACGTCCATCCTAAAGCCAACACCTAAAGCCTACACCTAAAGACCCATCAAGTCAACGCCTATCTTAAAGTTTAAACA‑‑‑‑‑‑‑‑‑‑‑‑‑TAAAGACCAGACCTAAAGACCAGACCTAAAGACACTACATAAAGACCAGACCTAAAGACGCCTTGTTGTTAGCCATAAAGTGATAAcc                                            <  1:194570/301‑1 (MQ=255)`  
                                                                                     `ccTTCGGTTGACCTTGAGGGTTCCCTAAGGGTTGGGGATGACCCTTGGGTTTGTCTTTGGGTGTTACCTTGAGTGTCTCTCTGTG‑T‑CC‑CTATCTGTAACAGTCTCCTAAAGTATCCTCCTAAAGTCACCTCCTAACGTCCATCCTAAAGCCAACACCTAAAGCCTACACCTAAAGACCCATCAAGTCAACGCCTATCTTAAAGTTTAAACA‑‑‑‑‑‑‑‑‑‑‑‑‑TAAAGACCAGACCTAAAGACCAGACCTAAAGACACTACATAAAGACCAGACCTAAAGACGCCTTGTTGTTAGCCATAAAGTGATAACCtt                                          >  1:78284/1‑301 (MQ=255)`  
                                                                                        `tCGGTTGACCTTGAGGGTTCCCTAAGGGTTGGGGATGACCCTTGGGTTTGTCTTTGGGTGTTACCTTGAGTGTCTATCTGTG‑T‑CC‑C‑ATCTGTTACAGTCTCCTAAAGTATCCTCCTAAAGTCACCTCCTAACGTCCATCCTAAAGCCAACACCTAAAGCCTACACCTAAAGACCCATCAAGTCAACGCCTATCTTAAAGTTTAAATA‑‑‑‑‑‑‑‑‑‑‑‑‑TAAAGACCAGACCTAAAGACCAGACCTAAAGACACTACATAAAGACCAGACCTAAAGACGCCTtgctgtctattatacaaatctccgagcccac                                      >  1:277204/1‑276 (MQ=38)`  
                                                                                         `gatgtgtagaagagacgtCCCTAAGGGTTGGGGATGACCCTTGGGTTTGTCTTTGGGTGTTACCTTGAGTGTCTCTCTGTG‑T‑CC‑C‑ATCTGTTACAGTCTCCTAAAGTATCCTCCTAAAGTCACCTCCTAACGTCCATCCTAAAGCCAACACCTAAAGCCTACACCTAAAGACCCATCAAGTCAACGCCTATCTTAAAGTTTAAACA‑‑‑‑‑‑‑‑‑‑‑‑‑TAAAGACCAGACCTAAAGACCAGACCGAAAGACACTACATAAAGACCAGACCTAAAGACGCCTTGTTGTTAGCCATAAAGTGATAACCTTTAATc                                     <  1:149856/284‑1 (MQ=255)`  
                                                                                         `cGGTTTACCTTGAGGTTTCCCTAAGGGTTGGGGATGACCCTTGGGTTTGGCTTTGGGTGTTACCTTGAGTGTCTCTCTGTG‑T‑CC‑C‑ATCTGTTACAGTCTCCTAAAGTATCCTCCTAAAGTCACCTCCTAACGTCCATCCTAAAGCCAACACCTAAAGCCTACACCTAAAGACCCATCAAGTCAACGCCTATCTTAAAGTTTAAACA‑‑‑‑‑‑‑‑‑‑‑‑‑TAAAGACCAGACCTAAAGACCAGACCTAAAGACACTACATAAAGACCAGACCTAAAGACGCCTTGTTGTTAGCCATAAAGTGATAACCTTTAATc                                     <  1:226573/301‑1 (MQ=255)`  
                                                                                         `cGGTTGACCGTGAGGTTTCCCAGAGGGGTGGGGATTACCCTTGGGTTTGTATTTGGGTGTTACCTTACGTGTTTCTCTGTG‑T‑AA‑C‑ATCTGATACAGTCGCCTAAAGCAGCCTCCTCGAGGCACCTCCTAACGTCTATGCTAAAGCCAACACCTACAGCCTAGACATAAAGACCCACCAGGGCAACGCCTATCTTAAAGTTTAAACA‑‑‑‑‑‑‑‑‑‑‑‑‑TAAAGACCAGACCTAAAGACCAGGCCGAAAGACACTAAATAAAGACCAGACCTAAAGACGCCTTGTTGTTAGCCATAAAGTGATAACCTTTAATc                                     <  1:201426/301‑1 (MQ=255)`  
                                                                                          `tgTTGACCGTGAGGGTTCCCTAAGGGTTGGGGATGACCCTTGGGTTTGTCTTTGGGTGTTACCTTGAGTGTCTCTCTGTG‑T‑CA‑CTATCTTTAACAGTCTCCTAAAGTATCCTCCTAAAGTCACCTCCTAACGTCCATCCTAAAGACAACACCTAAAGCCTACACCTAAAGACCCATCAAGTCAACGCCTATCTTAAAGTTTAAACA‑‑‑‑‑‑‑‑‑‑‑‑‑TAAAGACCAGACCTAAAGACCAGACCTAAAGACACTACATAAAGACCAGACCTAAAGACGCCTTGTTGTTAGCCATAAAGTGATAACCTTTAATc                                     <  1:100116/300‑1 (MQ=255)`  
                                                                                          `gggtGACCTTGAGTGTTCCCTAAGGGTTTGGGATGAACCTTGGGTTTGTCTTTGGGTGTTACCTTGAGTGGCTCTCTGTG‑T‑CC‑CTATCTGTAACAGTCTACTAAAGTATCCTACTAAAGTCACCTCCTAACGTCCATCCTAAAGCCAACACCTAAAGCCTACACCTAAAGACCCATCAAGTCAACGCCTATCTTAAAGTTTAAACA‑‑‑‑‑‑‑‑‑‑‑‑‑TAAAGACCAGACCTAAAGACCAGACCTAAAGACACTACATAAAGACCAGACCTAAAGACGCCTTGTTGTTAGCCATAAAGTGATAACCTTTAATc                                     <  1:310341/298‑1 (MQ=255)`  
                                                                                          `ggTTGACCTTGATGGTTCCCTAAGGGTTTGGGATGACCCTTTGGGTTGTCTTTGGGTGTTACCTTGAGTGTCTCTCTGTG‑T‑CC‑CTATCTGTAACAGTCTCCTAAAGTATCCTCCTAAAGTCACCTCCTAACGTCCATCCTAAAGCCAACACCTAAAGCCTACACCTAAAGACCCATCAAGTCAACGCCTATCTTAAAGTTTAAACA‑‑‑‑‑‑‑‑‑‑‑‑‑TAAAGACCAGACCTAAAGACCAGACCTAAAGACACTACATAAAGACCAGACCTAAAGACGCCTTGTTGTTAGCCATAAAGTGATAACCTTTAATc                                     <  1:300402/301‑1 (MQ=255)`  
                                                                                          `ggTTGACCTTGAGGGTTCCCTAAGGGTTGTGGATGACCCTTGGGTTTGTCTTTGGGTGTTACCTTGAGTGTCTCTCTGTG‑T‑CC‑CTATCTG‑TACAGTCTCCTAAAGTATCCTCCTAAAGTCACCTCCTAACGTCCATCCTAAAGCCAACACCTAAAGCCTACACCTAAAGACCCATCAAGTCAACGCCTATCTTAAAGTTTAAACA‑‑‑‑‑‑‑‑‑‑‑‑‑TAAAGACCAGACCTAAAGACCAGACCTAAAGACACTACATAAAGACCAGACCTAAAGACGCCTTGTTGTTAGCCATAAAGTGATAACCTTTAATCa                                    <  1:182873/301‑1 (MQ=255)`  
                                                                                          `ggTGGACCTTGAGGGTTCCCTAAGGGTTGGGGATGACCCTTGGGTTTGTCTTTGGGTGTTACCTTGAGTGTCTCTCTGTG‑TCCC‑CTATCAGTTACAGTCTCCTAAAGTATCCTCCTAAAGTCACCTCCTAACGTCCATCCGAAAGCCAACACCTAAAGCCTACACCTAAAGACCCATCAAGTCAACGCCTATCTTAAAGTTTAAACA‑‑‑‑‑‑‑‑‑‑‑‑‑TAAAGACCAGACCTAAAGCCCAGACCTAAAGACACTACATAAAGACCAGACCTAAAGACGCCTTGTTGTTAGCCATAAAGTGATAACCTTTAAt                                      <  1:136461/301‑1 (MQ=255)`  
                                                                                                `ccTTGAGGGTTCCCTAAGGGTTGGTGATGACCCTTGGGTTTGTCTTTGGGTGTTACCTTGAGTGTCTCTCTGTG‑T‑CC‑C‑ATCTGTTACAGTCTCCTAAAGTATCCTCCTAAAGTCACCTCCTAACGTCCATCCTAAAGCCAACACCTAAAGCCTACACCTAAAGACCCATCAAGTCAACGCCTATCTTAAAGTTTAAACA‑‑‑‑‑‑‑‑‑‑‑‑‑TAAAGACCAGACCTAAATACCAGACCTAAAGACACTACATAAAGACCAGACCTAAAGACGCCTTGTTGTTAGCCATAAAGTGATAACCTTTAATCATTGTCt                              <  1:139170/301‑1 (MQ=255)`  
                                                                                                     `aGGGTTCCCTAAGACTTGGCGATGTCCCTTTGGTTTGTCTTTCAGTGTTCCCTTGATTGTCTCTCTGTG‑T‑CC‑CTATCCG‑TACACTCTCCTAAAATAGCCTCCTAAAGTCACCTCCTAACGTCCATCCCAAAGCCACCACCTAAAGCCTACACATAAAGACCCATCAAGTCAACGCCTATCTTAAAGTTTAAACA‑‑‑‑‑‑‑‑‑‑‑‑‑TAAAGACCAGACCTAAAGACCAGACCTAAAGACACTACATAAAGACCAGACCTAAAGACGCCTTGTTGTTAGCCATAAAGTGATAACCTTTAATCATTGTCTttatt                         <  1:62798/301‑1 (MQ=255)`  
                                                                                                          `tCCCTTAGGGTTGGGTATGACCCTTGGGTTTGTCTTTGGGTGTTACCTTGAGTGTCTCTCTGTG‑T‑CC‑CTATCTG‑TACAGTCTCCTAAAGTATCCTCCTAAAGTCACCTCCTAACGTCCATCCTAAAGCCAACACCTAAAGCCTACACCTAAAGACCCATCAAGTCAACGCCTATCTTAAAGTTTAAACA‑‑‑‑‑‑‑‑‑‑‑‑‑TAAAGACCAGACCTAAAGACCAGACCTAAAGACACTACATAAAGACCAGACCTAAAGACGCCTTGTTGTTAGCCATAAAGTGATAACCTTTAATCATTGTCTTTATTAATAc                    <  1:29114/301‑1 (MQ=255)`  
                                                                                                          `tCCCTAAGGGTTGGGGATGACCCTTGGGTTTGTCTTTGGGTGTTACCTTGAGTGTCTCTCTGTG‑T‑CC‑CTATCTG‑TACAGTCTCCTAAAGTATCCTCCTAAAGTCACCTCCTAACGTCCATCCTAAAGCCAACACCTAAAGCCTACACCTAAAGACCCATCAAGTCAACGCCTATCTTAAAGTTTAAACA‑‑‑‑‑‑‑‑‑‑‑‑‑TAAAGACCAGACCTAAAGACCAGACCTAAAGACACTACATAAAGACCAGACCTAAAGACGCCTTGTTGTTAGCCATAAAGTGATAACCTTTAATCATTGTCTTTATTAATAc                    <  1:285546/301‑1 (MQ=255)`  
                                                                                                          `gtataagatacaggggATGACCCTTGGGTTTGTCTTTGGGTGTTACCTTGAGTGTCTCTCTGTG‑T‑CC‑CTATCTG‑TACAGTCTACTAAAGTATCCTCCTAAAGTCACCTCCTAACGTCCATCCTAAAGCCAACACCTAAAGCCTACACCTAAAGACCCATCAAGTCAACGCCTATCTTAAAGTTTAAACA‑‑‑‑‑‑‑‑‑‑‑‑‑TAAAGACCAGACCTAAAGACCAGACCTAAAGACACTACATAAAGACCAGACCTAAAGACGCCTTGTTGTTAGCCATAAAGTGATAACCTTTAATCATTGTCTTTATTAATAc                    <  1:65185/289‑1 (MQ=255)`  
                                                                                                           `cccTAAGGGTTGGGGATTAACCTTGGGTTTGTCTTTGGGTGTTACCTTGAGTGTCTCTCTGTG‑T‑CC‑CTATCTGTAACAGTCTCCTAAAGTATCCTCCTAAAGTCACCTCCTAACGTCCATCCTAAAGCCAACACCTAAAGCCTACACCTAAAGACCCATCAAGTCAACGCCTATCTTAAAGTTTAAACA‑‑‑‑‑‑‑‑‑‑‑‑‑TAAAGACCAGACATAAAGACCAGACCTAAAGACACTACATAAAGACCAGACCTAAAGACGCCTTGTTGTTAGCCATAAAGTGATAACCTTTAATCATTGTCTTTATTAATAc                    <  1:162690/301‑1 (MQ=255)`  
                                                                                                           `cccTAAGGGTTGGGGATGACCCTTGGGTTTGTCTTTGGGTGTTACCTTGAGTGTCTCTCTGTG‑T‑CC‑CTATCTGTAACAGTCTCCTAAAGTATCCTCCTAAAGTCACCTCCTAACGTCCATCCTAAAGCCAACACCTAAAGCCTACACCTAAAGACCCATCAAGTCAACGCCTATCTTAAAGTTTAAACA‑‑‑‑‑‑‑‑‑‑‑‑‑TAAAGACCAGACCTAAAGACCAGACCTAAAGACACTACATAAAGACCAGACCTAAAGACGCCTTGTTGTTAGCCATAAAGTGATAACCTTTAATCATTGTCTTTATTAATaa                    >  1:248413/1‑300 (MQ=255)`  
                                                                                                           `cccTAAGGGTTGGGGATGACCCTTGGGTTTGTCTTTGGGTGTTACCTTGAGTGTCTCTCTGTG‑T‑CC‑CTATCTG‑TACAGTCTCCTAAAGTATCCTCCTAAAGTCACCTCCTAACGTCCATCCTAAAGCCAACACCTAAAGCCTACACCTAAAGACCCATCAAGTCAACGCCTATCTTAAAGTTTAAACA‑‑‑‑‑‑‑‑‑‑‑‑‑TAAAGACCAGACCTAAAGACCAGACCTAAAGACACTACATAAAGACCAGACCTAAAGACGCCTTGTTGTTAGCCATAAAGTGATAACCTTTAATCATTgctgtctcttataca                   >  1:331423/1‑287 (MQ=255)`  
                                                                                                           `cccTAAGGGTTGGGGAGGACCCTTGGGTTTGTCTTTGGGTGTTACCTTGAGTGTCTTTCTGTG‑T‑CC‑CTATCTGTAACAGTCTCCTAAAGTATCCTCCTAAAGTCACCTCCTAACGTCCAGCCTAAAGCCAACACATAAAGCCTACACCTAAAGACCCATCAAGTCAACGCCTATCTTAAAGTTTAAACA‑‑‑‑‑‑‑‑‑‑‑‑‑TAAAGACCAGACCTAAAGACCAGACCTAAAGACACTACATAAAGACCAGACCTAAAGACGCCTTGTTGTTAGCCATAAAGTGATAACCTTTAATCATTGTCTTTATTAATAc                    <  1:155800/301‑1 (MQ=255)`  
                                                                                                           `cccTAAGGATTGGCGATGACCCTTTGGTTTGTCTTTGAGTGTTACCTTGAGTGTCTCTCTGTG‑T‑CC‑CTAGCTGTAACAGTCTCCTAAAGTATCCTCCTAAAGTCACCTCCTAACGTCCATCCTAAAGCCAACACCTAAAGCCTACGCCTAAAGACCCATCAAGTCAACGCCTATCTTAAAGTTTAAACA‑‑‑‑‑‑‑‑‑‑‑‑‑TAAAGACCAGACCTAAAGACCAGGCCTAAAGACACTACATAAAGACCAGACCTAAAGACGCCTTGTTGTTAGCCATAAAGTGATAACCTTTAATCATTGTCTTTATTAATAc                    <  1:47206/301‑1 (MQ=255)`  
                                                                                                            `ccTAAGGGTTGGGGATGACCCTTGGGTTTGTCTTTGGGTGTTACCTTGAGTGTCTCTCTGTG‑T‑CC‑CTATCTGTAACAGTCTCCTAAAGTATCCTCCTAAAGTCACCTCCTAACGTCAATCCTAAAGCCAACACCTAAAGCCTACACCTAAAGACCCATCAAGTCAACGCCTATCTTAAAGTTTAAACA‑‑‑‑‑‑‑‑‑‑‑‑‑TAAAGACCAGACCTAAAGACCAGACCTAACGACACTACATAAAGACCAGACCTCAAGACGCCTTGTTGTTAGCCATAAAGTGATAACCTTTAATCATTGTCTTTATTAATACa                   >  1:83812/1‑301 (MQ=255)`  
                                                                                                             `cTAAGGGTTGGGGATGACCCTTGGGTTTGTCTTTGGGTGTTACCTTGAGTGTCTCTCTGTG‑TCCC‑CTATCTGTTACAGTCTCCTAAAGTATCCTCCTAAAGTCACCTCCTAACGTCCATCCTAAAGCCAACACCTAAAGCCTACACCTAAAGACCCATCAAGTCAACGCCTATCTTAAAGTTTAAACA‑‑‑‑‑‑‑‑‑‑‑‑‑TAAAGACCAGACCTAAAGACCAGACCTAAAGACACTACATAAAGACCAGACCTAAAGACGCCTTGTTGTTAGCCATAAAGTGATAACCTTTAATCATTGTCTTTATTAATACa                   >  1:274317/1‑301 (MQ=255)`  
                                                                                                                 `gggTTGGGGATTACCCTTGGGTTTGTCTTTGGGTGTTACCTTGAGTGTCTCTCTGTG‑T‑CC‑CTATCTGTAACAGTCTCCTAAAGTATCCTCCTAAAGTCACCTCCTAACGTCCATCCTAAAGCCAACACCTAAAGCCTACACCTAAAGACCCATCAAGTCAACGCCTATCTTAAAGTTTAAACA‑‑‑‑‑‑‑‑‑‑‑‑‑TAAAGACCAGACCTAAAGACCAGACCTAAAGACACTACATAAAGACCAGACCTAAAGACGCCTTGTTGTTAGCCATAAAGTGATAACCTTTAATCATTGTCTTTATTAATACAACTCa              >  1:271544/1‑301 (MQ=255)`  
                                                                                                                    `ttGGGGATGAACATTGGGTTGTGCTTTTGGTGTTACCTTGAGTGTCTCTCTGTG‑TCCC‑CTATCTGTTACAGTCTCCTAAAGTATCCTCCTAAAGTAACCTCCTAACGTCCATCCTAAAGCCAACACCTAAAGCATACACCTAAAGACCCATCAAGTCAACGCCTATCTTAAAGTTTAAACATAAAGACCAGACCTAAAGACCAGACCTAAAGACCAGACCTAAAGACACTACATAAAGACCAGACCTAAAGACGCCTTGTTGTTAGCCATAAAGTGATAACCTTTAATCATTGTCTttatt                         <  1:116029/301‑1 (MQ=255)`  
                                                                                                                         `gttGAACCTTGGGTTTGTCTTTGGGTTTTACCTTGAGTGTCTCTCTGTG‑T‑CC‑CTATCTGTAACAGTCTCCTAAAGTATCCTCCTAAAGTCACCTCCTAACGTCCATCCTAAAGCCAACACCTAAAGCCTACACCTAAAGACCCATCAAGTCAACGCCTATCTTAAAGTTTAAACA‑‑‑‑‑‑‑‑‑‑‑‑‑TAAAGACCAGACCTAAAGACCAGACCTAAAGACACTACATAAAGACCAGACCTAAAGACGCCTTGTTGTTAGCCATAAAGTGATAACCTTTAATCATTGTCTTTATTAATACAACTCACTATAAgg      <  1:132393/299‑1 (MQ=255)`  
                                                                                                                         `gATGAACCTTGGGTTTGTCTTTGGGTGTTACCTTGAGTGTCTCTCTGGG‑T‑CC‑CTATCTGTAACAGTCTCCTAAAGTATCCTCCTAAAGTCACCTCCTAACGTCCATCCTAAAGCCAACACCTAAAGCCTACACCTAAAGACCCATCAAGTCAACGCCTATCTTAAAGTTTAAACA‑‑‑‑‑‑‑‑‑‑‑‑‑TAAAGACCAGACCTAAAGACCAGACCTAAAGACACTACATAAAGACCAGACCTAAAGACGCCTTGTTGTTAGCCATAAAGTGATAACCTTTAATCATTGTCTTTATTAATACAACTCACTATAAgg      <  1:280709/301‑1 (MQ=255)`  
                                                                                                                          `aTGACCCTTTGGTTTGTCTTTGTGTCTTACCTTGAGTGTCTCTCTGTT‑G‑CC‑CTATCCGTAACAGTCTCCTAAAGTATCCTCCTAAAGTCACCTCCTACCCTCCATCCTAAAGCCAACACCTAAAGCCTACACCTAAAGACCCATCAAGTCAACGCCTATCTTAAAGTTTAAACA‑‑‑‑‑‑‑‑‑‑‑‑‑TAAAGACCAGACCTAAAGACCAGACCTAAAGACACTACATAAAGACCAGACCTAAAGACGCCTTGTTGTTAGCCATAAAGTGATAACCTTTAATCATTGTCTTTATTAATACAACTCACTATAAGga     <  1:92585/301‑1 (MQ=255)`  
                                                                                                                          `aTGACCCTTGGGTTTGTCTTTGGGTGTTACCTTGAGTGTCTCTCTGTG‑T‑CC‑CTATCTGTAACAGTCTCCTAAAGTATCCTCCTAAAGTCACCTCCTAACGTCCATCCTAAAGCCAACACCTAAAGCCTACACCTAAAGACCCATCAAGTCAACGCCTATCTTAAAGTTTAAACA‑‑‑‑‑‑‑‑‑‑‑‑‑TAAAGACCAGACCTAAAGACCAGACCTAAAGACACTACATAAAGACCAGACCTAAAGACGCCTTGTTGTTAGCCATAAAGTGATAACCTTTAATCATTGTCTTTATTAATACAACTCACTATAAGga     <  1:106739/301‑1 (MQ=255)`  
                                                                                                                          `aTGACCCTTGGGTTTGTCTTTGGGTGTTACCTTGAGTGTCTATCTGTG‑T‑CC‑CTATCTGTAACAGTCTCCTAAAGTATCCTCCTAAAGTCACCTCCTAACGTCCATCCTAAAGCCAACACCTAAAGCCTACACCTAAAGACCCATCAAGTCAACGCCTATCTTAAAGTTTAAACA‑‑‑‑‑‑‑‑‑‑‑‑‑TAAAGACCAGACCTAAAGACCAGACCTAAAGACACTACATAAAGACCAGACCTAAAGACGCCTTGTTGTTAGCCATAAAGTGATAACCTTTAATCATTGTCTTTATTAATACAACTCACTATAAGga     <  1:185724/301‑1 (MQ=255)`  
                                                                                                                            `gACCCTTGGGTTTGTCTTTGGGTGTTACCTTGAGTGTCTCTCTGTG‑T‑CC‑C‑ATCTGTTACAGTCTCCTAAAGTATCCTCCTAAAGTCACCTCCTAACGTCCATCCTAAAGCCAACACCTAAAGCCTACACCTAAAGACCCATCAAGTCAACGCCTATCTTAAAGTTTAAACA‑‑‑‑‑‑‑‑‑‑‑‑‑TAAAGACCAGACCTAAAGACCAGACCTCAAGACACAACATAACGACCAGACCTAAAGACGCCTTGTTGTTAGCCATAAAGTGATAACCTTTAATCATTATCTTTATTAATACAACTCACTATaattcttt  >  1:189596/1‑295 (MQ=255)`  

                                                                                                                             `|`                                                                                                                                                                                                                                                                                                                              
`‑‑‑‑‑‑‑‑‑‑‑‑‑TCTCACAGTGTACGGACCTAAAGTT‑CCCCCATA‑GGGGGTACCTAAAGCCCAGCCAATCACCTAAAGTCAACCTTCGGTTGACCTTGAGGGTTCCCTAAGGGTTGGGGATGACCCTTGGGTTTGTCTTTGGGTGTTACCTTGAGTGTCTCTCTGTG‑T‑CC‑CTATCTGTTACAGTCTCCTAAAGTATCCTCCTAAAGTCACCTCCTAACGTCCATCCTAAAGCCAACACCTAAAGCCTACACCTAAAGACCCATCAAGTCAACGCCTATCTTAAAGTTTAAACA‑‑‑‑‑‑‑‑‑‑‑‑‑TAAAGACCAGACCTAAAGACCAGACCTAAAGACACTACATAAAGACCAGACCTAAAGACGCCTTGTTGTTAGCCATAAAGTGATAACCTTTAATCATTGTCTTTATTAATACAACTCACTATAAGGAGAG  >  NC_001604.1/1‑411`

  

Alignment Legend

Aligned base mismatch/match (shaded by quality score): `ATCG/ATCG` `< 3 ≤` `ATCG/ATCG` `< 11 ≤` `ATCG/ATCG` `< 23 ≤` `ATCG/ATCG` `< 34 ≤` `ATCG/ATCG` `< 38 ≤` `ATCG/ATCG`

Unaligned base: `atcg`    Masked matching base: `atcg`    Alignment gap: `‑`    Deleted base: `‑`
